# Perinatal determinants of neonatal hair glucocorticoid concentrations

**DOI:** 10.1016/j.psyneuen.2021.105223

**Published:** 2021-06

**Authors:** David Q. Stoye, Gemma Sullivan, Paola Galdi, Clemens Kirschbaum, Gillian J. Lamb, Gill S. Black, Margaret J. Evans, James P. Boardman, Rebecca M. Reynolds

**Affiliations:** a MRC Centre for Reproductive Health, University of Edinburgh, Edinburgh, UK; b Faculty of Psychology, Technische Universität Dresden, Dresden, Germany; c Department of Pathology, Royal Infirmary of Edinburgh, Edinburgh, UK; d Centre for Clinical Brain Sciences, University of Edinburgh, Edinburgh, UK; e Centre for Cardiovascular Science, University of Edinburgh, UK

**Keywords:** Cortisol, Hypothalamus–pituitary–adrenal axis, Parturition, Chorioamnionitis, Preterm birth, Stress

## Abstract

Adult hair glucocorticoid concentrations reflect months of hypothalamic-pituitary-adrenal axis activity. However, little is known about the determinants of neonatal hair glucocorticoids. We tested associations between perinatal exposures and neonatal hair glucocorticoids. Cortisol and cortisone were measured by LC-MS/MS in paired maternal and infant hair samples collected within 10 days of birth (n = 49 term, n = 47 preterm), with neonatal samples collected at 6-weeks in n = 54 preterm infants. We demonstrate cortisol accumulation in hair increases with fetal maturity, with hair cortisol being higher in term than preterm born infants after delivery (median 401 vs 106 pg/mg; p < 0.001). In term born infants, neonatal hair cortisol is positively associated with maternal hair cortisol concentration (β = 0.240, p = 0.045) and negatively associated with birthweight z-score (β = −0.340, p = 0.006). Additionally, being born without maternal labour is associated with lower hair cortisol concentrations (β = −0.489, p < 0.001) and a lower ratio of cortisol to cortisone (β = −0.484, p = 0.001). In preterm infants, histological chorioamnionitis is associated with a higher cortisol to cortisone ratio in hair (β = 0.459, p = 0.001). In samples collected 6 weeks after preterm birth, hair cortisol concentration is associated with cortisol hair concentrations measured after birth (β = 0.523, p < 0.001), chorioamnionitis (β = 0.250, p = 0.049) and postnatal exposures including intravenous hydrocortisone therapy (β = 0.343, p < 0.007) and neonatal sepsis (β = 0.290, p = 0.017). In summary, neonatal hair cortisol is associated with birth gestation, maternal hair cortisol concentration and fetal growth. Additionally, exposures at delivery are important determinants of hair cortisol, and should be considered in the design of future research investigating how neonatal hair cortisol relates to prenatal exposures or fetal development.

## Highlights

• Neonatal hair cortisol is higher after term compared to preterm birth.
• Neonatal hair cortisol reflects fetal growth, maternal cortisol and birth exposures.
• Hair sampled at 6 weeks reflects postnatal exposures in preterm infants.

## 1 Introduction

There is a growing evidence from human observational studies that maternal health and wellbeing influence fetal growth, and offspring health across the life-course (Hoffman et al., 2017a). Fetal cortisol exposure is one of the key mechanisms hypothesized to mediate these relationships (Moisiadis and Matthews, 2014b). During healthy pregnancies fetal exposure to cortisol is tightly regulated, in part by the activity of the enzyme 11β -hydroxysteroid dehydrogenase type 2 (11β-HSD2) in the placenta, which converts cortisol to inert cortisone. Whilst maternal and fetal hypothalamic-pituitary-adrenal (HPA) axes are upregulated before birth in healthy term pregnancies, and increased cortisol helps prime the fetus for life outside the womb (Fowden et al., 1998), excess exposure to developing tissues may reduce fetal growth and adversely affect long term health (Reynolds, 2013).

Directly measuring fetal cortisol exposure is challenging (Meyer and Novak, 2020). Sampling of fetal blood or amniotic fluid, involves invasive procedures that risk causing harm to mother and fetus (Enzensberger et al., 2012), and postnatal cortisol concentrations measured in cord blood are confounded by factors at delivery (Stirrat et al., 2017). Instead, studies have typically relied on indirect estimates thought to represent fetal cortisol exposure including maternal psychosocial stress, liquorice exposure, exogenous steroid administration or endogenous cortisol concentrations in pregnancy (Zijlmans et al., 2015).

Postnatal endogenous cortisol exposure may also impact the development of infants born preterm. Whilst preterm infants’ capacity to produce cortisol in the neonatal period is limited in comparison adults, demonstrated through studies of dynamic HPA axis function (Finken et al., 2016), serum cortisol concentrations in the preterm population in the weeks after birth are up to seven times higher than the low cortisol concentrations of fetuses at comparative gestational ages (Gitau et al., 1998, Glover et al., 2005, Jobe, 2009). Furthermore, preterm infants are at increased risk of later adverse metabolic (Parkinson et al., 2013) and neurodevelopmental (Johnson and Marlow, 2017) outcomes, phenotypes observed in preclinical models following excess perinatal glucocorticoid exposure (Moisiadis and Matthews, 2014a). Whilst there is evidence that noxious environmental stimuli (Vinall and Grunau, 2014) and postnatal high dose dexamethasone adversely affect neurodevelopment (Baud, 2004), little is known about the importance of cortisol exposure for a preterm population (Stoye et al., 2020). Characterisation of cortisol concentrations through traditional methods, including saliva and serum, have proven challenging in this population, as saliva volumes are frequently insufficient for analysis (Mörelius et al., 2016), and infants have an insufficient circulatory reserve to allow for repeated blood sampling.

Hair sampling is a candidate method through which to assess both prenatal and postnatal cortisol exposure (Meyer and Novak, 2020). In adults, including pregnant women, hair cortisol concentration is validated as a long-term marker of HPA axis activity with moderate to strong correlations with repeated measures of saliva cortisol (D'Anna-Hernandez et al., 2011, Short et al., 2016). Furthermore, in adults the demographic correlates of hair cortisol have been established in large observational studies (Stalder et al., 2017). In contrast, relatively little is known about the determinants of neonatal hair cortisol concentrations. Reports of whether neonatal hair cortisol reflects maternal cortisol in pregnancy are conflicting, with previous studies reporting either positive (Hollanders et al., 2017) or no associations (Hoffman et al., 2017b, Schury et al., 2017). Studies assessing determinants of neonatal hair cortisol in both humans and primate models have typically focused on maternal factors experienced throughout pregnancy, such as psychosocial stress (Kapoor et al., 2016, Romero-Gonzalez et al., 2018, van der Voorn et al., 2018), and with the exception of one study (Hollanders et al., 2017), have not reported associations between hair cortisol and exposures at the time of delivery. Additionally, whilst previous studies have demonstrated positive associations between birth gestation and hair cortisol concentration, these have predominantly included infants born at term or late preterm (Hoffman et al., 2017b, Hollanders et al., 2017).

The primary aim of this study was to assess determinants of neonatal hair glucocorticoid concentrations (cortisol and cortisone) sampled within the first 10 days of birth in term and very preterm infants. We hypothesised that hair glucocorticoids would be higher in neonates born at term (≥ 37 weeks’ gestation), compared to infants born very preterm (≤ 32 weeks’ gestation), reflecting increases in maternal and fetal HPA axes across pregnancy, and that glucocorticoid concentrations in neonatal hair would be positively associated with maternal cortisol concentrations, and negatively associated with fetal growth. Additionally, we tested whether infant hair glucocorticoid concentrations reflect exposures at the time of delivery. Secondary aims were to test associations between perinatal exposures and hair glucocorticoids of preterm infants assessed 6 weeks after birth, and maternal postnatal hair glucocorticoids after delivery.

## 2 Material and methods

### 2.1 Participants

The Stress Response Systems in Mothers and Preterm Infants study is a longitudinal cohort assessing the role of glucocorticoids in the early life programming of health and disease (Stoye et al., 2020). Participants were mother-term (≥ 37 weeks’ gestational age) and mother-preterm dyads (≤ 32 weeks’) with births at the Royal Infirmary, Edinburgh between March 2018 and August 2019. The study design included informed consent that was either antenatal (routine antenatal clinics, ahead of planned caesarean sections, or presentation with threatened preterm birth), or postnatal if there was not sufficient time for informed consent before the onset of labour. Exclusion criteria were regular maternal corticosteroid use in pregnancy, and congenital fetal abnormality or chromosomal abnormality. Ethical approval was granted by South East Scotland 01 Regional Ethics Committee (18/SS/0006), and all mothers gave written informed consent.

### 2.2 Hair cortisol and cortisone measurement

Hair was sampled from mother-infant dyads within the first 10 days of birth (Time-point 1). In mothers, hair was cut close to the scalp, at the posterior vertex. In neonates, hair was typically cut using scissors from the nape of the neck. However, due to sparse hair growth in some participants sampling was not limited to this region. In preterm infants, hair sampling was repeated at 6 weeks after birth (Time-point 2). Hair was stored in aluminium foil at − 20 C, and cortisol and cortisone concentrations were measured by liquid chromatography-tandem mass spectrometry (LC-MS/MS), at the Technische Universität, Dresden using an established method (Gao et al., 2013). In mothers the 3 cm of hair nearest the scalp was analysed, representing the last three months of pregnancy. In neonates, the whole hair segment was assessed at time-point 1, with analysis restricted to the 2 cm nearest the scalp assessed at time-point 2.

### 2.3 Assessment of perinatal exposures

Participant demographic and medical information was collected by completion of a maternal questionnaire and review of medical records by a trained research nurse or doctor. Maternal labour status was categorised according to whether mothers went into labour spontaneously, were induced, or gave birth by elective caesarean without having been in labour. Neonatal sepsis was defined as a positive blood culture with a pathogenic organism and/or physician decision to treat with intravenous antibiotics for ≥ 5 days based on abnormal clinical signs and laboratory tests, at any time in the neonatal period. Birthweight z-score was calculated according to International Fetal and Newborn Growth Consortium for the 21st Century (INTERGROWTH-21st) standards (Villar et al., 2014). Placentas collected after delivery were examined by an experienced perinatal pathologist (MJE), and histological chorioamnionitis was defined as the presence of an inflammatory response in the placental membranes (Sullivan et al., 2020).

During the study period, there was a change in practice at the neonatal unit, Royal Infirmary, Edinburgh, whereby infants born < 28 weeks were given prophylactic hydrocortisone, as per a regimen set out in the PREMILOC study (Baud et al., 2016). This meant that 11 of 15 infants born < 28 weeks’ gestation in the study received an early postnatal course of intravenous hydrocortisone hemisuccinate at 1 mg/kg per day for 7 days, followed by doses of 0·5 mg/kg per day for 3 days.

### 2.4 Statistical analysis

Analysis was conducted using SPSS 25 Armonk, NY: IBM Corp. Participant demographics are described as mean ± SD if normally distributed and mean (range) if skewed.

Hair glucocorticoid outcomes of interest were:

1) Hair cortisol concentration
2) Hair cortisone concentration
3) The ratio of hair cortisol to cortisone concentrations

A single maternal hair sample, and single neonatal hair samples at each of time-points 1 and 2, with outlying cortisol concentrations > 3 standard deviations above the mean, were excluded from analysis. Remaining hair cortisol concentrations, cortisone concentrations, and cortisol to cortisone ratios were positively skewed and normalised through log-10 transformation.

Hair glucocorticoid concentrations in maternal and neonatal hair are presented as median (interquartile range) in pg/mg. Independent *t*-tests were conducted to assess if neonatal and maternal hair cortisol concentrations sampled within 10 days of birth (time-point 1) differed between term and preterm dyads. Paired *t*-tests were conducted to assess if hair glucocorticoids in preterm infants changed between the first 10 days of birth (time-point 1) and 6 weeks after birth (time-point 2). Associations between maternal hair glucocorticoid concentrations, infant hair glucocorticoid concentrations and perinatal exposures were tested through two regression models: a simple unadjusted model testing univariate associations; and a mutually adjusted model including variables with hair glucocorticoid concentrations or ratios with a p-value < 0.1 in the univariate analysis. Regression analysis was stratified according to whether infants were born at term or preterm, and results are presented as standardised beta coefficients (β). P-values < 0.05 are considered statistically significant.

## 3 Results

### 3.1 Demographics

Participant and sampling demographics are presented in Table 1. 93 mothers and 97 infants had hair sampled within 10 days of delivery. Additionally, 54 preterm infants had hair sampled 6 weeks after birth. Antenatal corticosteroids for threatened preterm delivery were given to all mothers of infants born preterm, and to none of the mothers who delivered at term.

**Table 1:** Participant and sampling demographics.

	Term group (≥ 37weeks GA)	Preterm group (32 ≤ weeks GA)
**Mothers, n**	n = 50	n = 43
Age (years)	34.1 ± 4.5	31.9 ± 5.9
Body mass index (kg/m^2^)	24.9 ± 4.3	25.5 ± 5.9
Tobacco smoked during pregnancy, n (%)	0 (0%)	7 (16%)
Received a single course of antenatal Steroids, n (%)	0 (0%)	43 (100%)
Labour onset, n (%)		
Spontaneous	26 (52%)	25 (58%)
Induced	15 (30%)	2 (5%)
Mode of Delivery, n (%)		
Spontaneous vaginal delivery	26 (52%)	17 (40%)
Instrumental delivery	8 (16%)	2 (5%)
Pre-labour caesarean section	9 (18%)	16 (37%)
In labour caesarean section	7 (14%)	8 (19%)
**Infant characteristics, n (%)**^a^	n = 49	n = 59
Hair collected at Time-points 1 and 2	0 (0%)	42 (71%)
Hair collected at Time-point 1 only	49 (100%)	5 (8%)
Hair collected at Time-point 2 only^b^	0 (0%)	12 (20%)
Birthweight (grams), mean (range)	3536 (2410, 4580)	1332 (454, 2380)
Birth weight z-score	0.5 ± 1.0	0.1 ± 1.1
Birth gestation (weeks), mean (range)	40.0 (37.6, 42.1)	29.4 (24.0, 32.0)
< 28 weeks gestation age at birth, n (%)	Not applicable	15 (25%)
Female, n (%)	25 (51%)	19 (32%)
Twin, n (%)	0 (0%)	18 (31%)
Exposure to histological chorioamnionitis, n (%)^c^	4 (16%)	21 (38%)
Received postnatal hydrocortisone, n (%)	0 (0%)	11 (19%)
Neonatal sepsis, n (%)	0 (0%)	9 (15%)
**Sample Characteristics**		
Maternal sample: Days after birth	2 (1–5)	4 (2–6)
Infant sample (time-point 1): Days after birth	2 (1–5)	4 (3–6)
Infant sample (time-point 2): Days after birth	Not applicable	44 (42–49)

Data are presented as mean ± SD if normally distributed, and mean (range) if skewed.

a In infants of mothers with hair sampled, hair was not collected in 1 term infant (whose parents did not consent for infant hair sampling, and 4 preterm participants (due to insufficient hair)).

b 12 preterm infants had hair sampled only at time-point 2 as parental consent for study entry was given after the first 10 days of life.

c N = 24 term placentas (n = 19 due to postnatal recruitment) and n = 3 preterm placentas where not assessed for chorioamnionitis

### 3.2 Hair glucocorticoid concentrations collected within 10 days of delivery (Time-point 1)

Glucocorticoid concentrations measured in maternal and infant hair samples are shown in Table 2. In hair samples collected with the first 10 days after delivery, concentrations of cortisol, cortisone and cortisol to cortisone ratios were all markedly higher in both preterm and term neonates compared to maternal samples. In term-dyads, median neonatal hair cortisol was 401 compared to 5.7 pg/mg in maternal hair, and the median ratio of hair cortisol to cortisone was 2.4 compared to 0.36. Hair cortisol, cortisone, and cortisol to cortisone ratios were also all higher in term compared to preterm infants (p < 0.001). Hair cortisol concentration was higher in mothers of term infants compared to the mothers of preterm infants, but the difference was modest (p = 0.045).

**Table 2:** Neonatal and maternal hair glucocorticoid concentrations and ratios. Median and IQR of hair glucocorticoid concentration in pg/mg.

		Term- Time-point 1 (< 10 days after delivery)	Preterm- Time-point 1 (< 10 days after delivery)	Preterm- Time-point 2 (6 weeks after delivery)
Maternal hair	Hair cortisol concentration	5.7 (3.4–13.6)**	5.0 (2.1–8.6)	NA
	Hair cortisone concentration	16.9 (11.4–25.3)*	15.0 (6.2–19.1)	NA
	Hair cortisol to cortisone ratio	0.36 (0.27–0.59)	0.35 (0.26–0.44)	NA
Neonatal hair	Hair cortisol concentration	401 (252–615)***	106 (66–164)	82 (55–169)
	Hair cortisone concentration	156 (135–192)***	65 (38–97)	94 (72–126)***
	Hair cortisol to cortisone ratio	2.4 (1.8–3.9)***	1.3 (1.0–2.4)	0.9 (0.6–2.0)***

In the term (time-point 1) column this represents results of term vs preterm postnatal hair glucocorticoids tested by independent t-test. In the preterm (time-point 2) column this represents results of preterm time-point 1 vs time-point 2 hair glucocorticoids tested by paired t-test.

* p-value < 0.1.

** p-value < 0.05.

*** p-value < 0.01.

In term infants, birthweight z-score was negatively associated with hair cortisol (β = −0.340, p = 0.006) and cortisol to cortisone ratio (β = −0.365 p = 0.005) (Table 3), and maternal and infant hair cortisol were positively associated (β = 0.240, p = 0.045). Hair glucocorticoid concentrations of preterm born infants were not associated with birthweight z-score or maternal glucocorticoids.

**Table 3:** Linear regression, testing associations of neonatal hair glucocorticoid concentrations, maternal glucocorticoid concentrations, birthweight z-score and exposures around delivery.

Exposure	Hair cortisol concentration		Hair cortisone concentration		Hair cortisol to cortisone ratio	
	Unadjusted model	Adjusted model	Unadjusted model	Adjusted model	Unadjusted model	Adjusted model
**Term Group**						
Corresponding maternal hair glucocorticoid	0.261*	0.240**	0.066	NA	0.066	NA
Birth gestation	0.257*	0.127	-0.015	NA	0.295**	0.157
Birthweight z-score	-0.287**	-0.340***	-0.023	NA	-0.304**	-0.365***
Labour						
Spontaneous	Ref	Ref	Ref		Ref	Ref
Induced	-0.324**	-0.302**	-0.292*	NA	-0.163	-0.124
No labour	-0.505***	-0.489***	-0.129	NA	-0.474***	-0.484***
**Preterm group**						
Corresponding maternal hair glucocorticoid	0.017	NA	-0.173	NA	0.215	NA
Birth gestation	0.425***	NA	0.354**	0.252*	0.166	NA
Birthweight z-score	0.108	NA	0.108	NA	0.026	NA
Labour						
No labour	-0.008	NA	-0.147	NA	0.125	NA
Chorioamnionitis	0.185	NA	-0.297**	-0.227	0.459***	NA

Where at least two variables had a p < 0.1 in unadjusted models these were included as covariates into a mutually adjusted regression model.

* p-value < 0.1.

** p-value < 0.05.

*** p-value < 0.01.

**Fig. 1:**
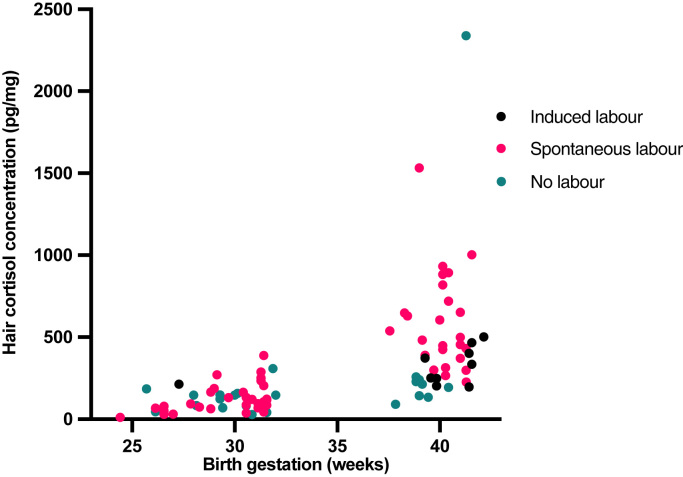
Infant Hair Cortisol (pg/mg), in samples collected within 10 days of delivery, labelled according to whether labour was spontaneous or induced, or infants were born by a caesarean section without labour. Being born without maternal labour was associated with lower hair cortisol concentrations (β = −0.489, p < 0.001).

**Fig. 2:**
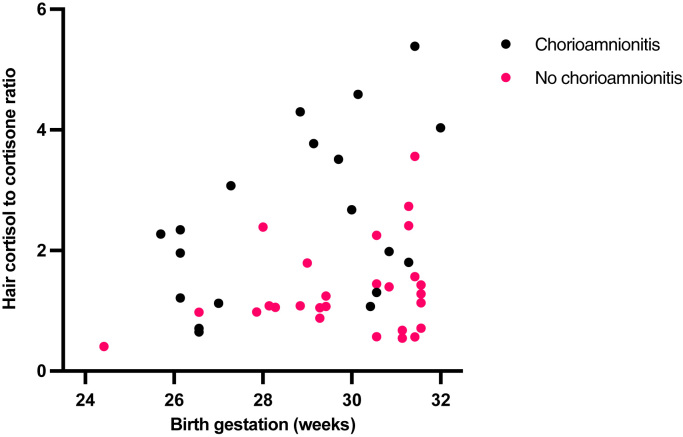
Infant hair cortisol to cortisone ratio, in samples collected within 10 days of delivery, according to whether there was histological chorioamnionitis. Histological chorioamnionitis was associated with a higher cortisol to cortisone ratio in hair (β = 0.459, p = 0.001).

Neonatal hair glucocorticoid concentrations were also associated with exposures around the time of delivery (Table 3). Term infants born to mothers who did not labour (β = −0.489, p-value < 0.001), and those born following induction of labour (β = −0.302, p = 0.018), had lower hair cortisol concentrations than infants of women who had spontaneous labour (Fig. 1). Birth following spontaneous labour compared to no labour (β = −0.484, p < 0.001), was also associated with a lower hair cortisol to cortisone ratio. In preterm infants, hair glucocorticoid concentrations did not differ according to labour or mode of delivery. However, histological chorioamnionitis was associated with a higher cortisol to cortisone ratio (β = 0.459, p = 0.001) in the hair of preterm infants (Fig. 2).

Maternal hair glucocorticoid concentrations were not associated with labour status, chorioamnionitis or infant birthweight z-score.

### 3.3 Hair glucocorticoid concentrations in preterm infants collected 6 weeks after delivery (Time-point 2)

In the 42 preterm infants with hair sampled on two occasions, hair cortisone increased (p = 0.009), the ratio of cortisol to cortisone decreased (p = 0.003), and there was no change in hair cortisol concentrations (p = 0.796) from early to 6 week samples (Table 2). There were positive associations between early and 6-week neonatal hair for cortisol (β = 0.523, p < 0.001) and cortisol to cortisone ratios (β = 0.574, p < 0.001), but not cortisone (β = −0.103, p = 0.517). Maternal hair glucocorticoids were not associated with infant 6-week hair glucocorticoids.

In adjusted models, chorioamnionitis was associated with hair cortisol concentration (β = 0.250, p = 0.049) and the ratio of cortisol to cortisone (β = 0.372, p = 0.008) (Table 4). Both neonatal sepsis (β = 0.290, p = 0.017) and postnatal hydrocortisone administration (β = 0.343, p = 0.007), were also positively associated with hair cortisol concentration. Birth gestation was negatively associated with hair cortisone concentration (β = −0.439, p = 0.012).

**Table 4:** Linear regression, testing associations of 6-week hair glucocorticoid concentrations with perinatal exposures.

	Standardised beta coefficients (β)					
Exposure	Hair cortisol		Hair cortisone		Hair cortisol to cortisone ratio	
	Unadjusted model	Adjusted model	Unadjusted model	Adjusted model	Unadjusted model	Adjusted model
Birth gestation	-0.156	NA	-0.464***	-0.439**	0.094	NA
Birthweight z-score	-0.037	NA	-0.239*	-0.197	0.092	NA
Chorioamnionitis	0.444***	0.250**	-0.027	NA	0.449***	0.372***
Neonatal sepsis	0.379***	0.290**	0.381***	0.181	0.171	NA
Postnatal hydrocortisone	0.442***	0.343***	0.237*	-0.070	0.311**	0.214

Mutually adjusted models included all exposures with a univariate association of p < 0.1.

* p-value < 0.1.

** p-value < 0.05.

*** p-value < 0.01.

## 4 Discussion

We collected hair in the early postnatal period from a cohort of mother-term and mother-preterm infant dyads, with additional hair sampling from preterm infants at 6 weeks. Hair cortisol, cortisone and the ratio of cortisol to cortisone measured in samples collected within 10 days of birth were all higher in infants compared to mothers, and in term compared to preterm infants. Hair glucocorticoid concentrations in neonatal samples collected within 10 days of birth were associated with maternal hair glucocorticoids, fetal growth and maternal labour. Hair glucocorticoids collected from preterm infants at 6 weeks were associated with both hair glucocorticoid concentrations after birth, and postnatal exposures including intravenous hydrocortisone therapy and neonatal sepsis. These findings allow inferences to be made about both the determinants of neonatal hair cortisol, and aspects of the maternal and fetal glucocorticoid regulation in the perinatal period.

Multiple features of neonatal hair cortisol are consistent with it being representative of cumulative cortisol exposure across the third trimester of pregnancy. Higher hair cortisol concentrations observed in term compared to preterm infants is in keeping with the expected physiological surge in fetal cortisol exposure during the third trimester (Fowden et al., 1998). The positive association between neonatal and maternal hair cortisol concentrations at term, previously demonstrated in one cohort (Hollanders et al., 2017), is consistent with studies showing associations between maternal and neonatal cortisol through other biological mediums, including cord blood (Gitau et al., 1998) and amniotic fluid (Baibazarova et al., 2013). The negative association between neonatal hair cortisol and birthweight z-score, whilst not previously observed in neonatal hair (Hollanders et al., 2017), is consistent with evidence generated from a wide range of preclinical and human observational models which show that prenatal cortisol exposure is associated with reduced fetal growth (Duthie and Reynolds, 2013).

This study also shows that neonatal hair cortisol reflects exposures at the time of delivery, including labour status and exposure to chorioamnionitis. Labour is a profound physiological stressor for both mothers and newborn infants, known to activate maternal and fetal HPA axes. This adds to evidence gathered from adults showing that whilst hair cortisol concentrations are markers of long term cortisol exposure, they reflect recent stressors most strongly (Kalliokoski et al., 2019). That hair cortisol concentrations are higher in infants of mothers who went into spontaneous labour, compared to those with an induced labour, is also intriguing, and could represent hypothalamic-pituitary adrenal axis upregulation in the weeks prior to spontaneous labour (Li et al., 2014).

Blunting of maternal and fetal stress responses by antenatal steroid therapy could potentially have contributed to the lower hair cortisol concentrations observed in preterm compared to term infants, and the lack of association between maternal labour and hair cortisol in preterm infants. A transient suppression of the HPA axis after antenatal steroid administration has been demonstrated through studies of fetal and maternal serum and cord blood (Waffarn and Davis, 2012). Fetal HPA axis suppression after antenatal corticosteroid therapy was observed in 18 of 22 studies included in a systematic review (Tegethoff et al., 2009), and maternal adrenal suppression has also been observed after repeated courses of antenatal corticosteroid therapy (Helal et al., 2000, McKenna et al., 2000). Differences in HPA axis activity between preterm infants exposed and unexposed to antenatal corticosteroids typically attenuate within two weeks of birth (Tegethoff et al., 2009), and mothers who receive a single course of antenatal corticosteroids for threatened preterm birth before 33 weeks and who go on to deliver at term, do not show HPA axis suppression at 38 weeks gestation (McKenna and Fisk, 2004). As all mothers in the preterm group received antenatal corticosteroids, this study cannot directly test the impact of antenatal corticosteroid therapy on hair glucocorticoid concentrations.

Neonatal hair glucocorticoid concentrations were more than 10-fold higher than maternal hair glucocorticoids, and this is consistent with previous studies measuring paired maternal and neonatal samples (Hoffman et al., 2017b, Hollanders et al., 2017, Kapoor et al., 2014, Schury et al., 2017). Little is known about why this is, and how cortisol is ultimately incorporated into neonatal hair. In adults, hair glucocorticoid concentrations largely reflect the incorporation of free cortisol from blood directly into the medulla, with smaller contributions made through sweat and sebum. In neonates, amniotic fluid has been hypothesised to be an additional determinant of neonatal hair cortisol, and multiple characteristics of our findings support this hypothesis.

First, neonatal hair has a high cortisol to cortisone ratio. In term born infants there was a cortisol to cortisone ratio of 2.4 compared to 0.36 in their mothers. Cortisol to cortisone ratios observed in neonatal hair are similar to those previously measured in amniotic fluid, in which cortisol to cortisone ratios have also been demonstrated to increase with increasing gestational age (Fencl et al., 1980).

Second, is the observation that preterm infant hair cortisol sampled at 6 weeks (median 82 pg/mg), is substantially lower than that of infants born at term (median 401 pg/mg). This difference in hair cortisol concentrations is unlikely to be a simple reflection of serum cortisol concentrations, as preterm infants have previously been shown to have higher serum cortisol than fetuses at comparative gestations (Gitau et al., 1998, Glover et al., 2005, Jobe, 2009), and is unlikely to reflect a unique ability of hair to uptake serum cortisol at a specific stage in hair development as these samples were only taken when participants were a few weeks apart in corrected gestational age. This builds on knowledge from previous studies that show a substantial drop-off in hair cortisol concentrations in both humans and other primate species, in the weeks after birth, but had not been able to exclude the possibility that this was related to the stage of development of the hair rather than time since exposure to amniotic fluid (Grant et al., 2017, Hollanders et al., 2017).

Alternative factors that could contribute to high hair cortisol concentrations and cortisol to cortisone ratios observed in neonatal hair include changes in regulation of systemic glucocorticoid metabolism and clearance, or local glucocorticoid metabolism by eccrine sweat glands, sebaceous glands and skin proximal to the hair follicle. However, fetuses have previously been demonstrated to have lower serum cortisol to cortisone ratios than adults making it unlikely that systemic glucocorticoid metabolism explains the patterns observed in neonatal hair (Fencl et al., 1980). To our knowledge 11β-HSD2 activity at sites proximal to the fetal hair follicle have not been investigated in the fetus.

If neonatal hair cortisol and cortisone concentrations are taken to be representative of amniotic fluid glucocorticoid concentrations this allows inferences to be drawn around why cortisol to cortisone ratios were highest in term participants who had a spontaneous labour, and higher in preterm infants exposed to chorioamnionitis compared to those without placental inflammation. Maternal serum and fetal urine are key sources of amniotic fluid glucocorticoids (Baibazarova et al., 2013). Additionally, the concentrations of specific glucocorticoids in amniotic fluid may also be influenced by enzymatic activity in the amnion, the membrane that contains amniotic fluid, which is rich in 11β-HSD1 and capable of converting cortisone to cortisol (Murphy, 1977). It has previously been demonstrated that amnion 11β-HSD1 is upregulated in chorioamnionitis, and speculated that upregulation of 11β-HSD1 might contribute to onset of labour (Braun et al., 2013, Chapman et al., 2013, Wang et al., 2018, Wang et al., 2020). This study adds to evidence that cortisone is increasingly regenerated to cortisol in chorioamnionitis and before spontaneous birth.

This is the first study to collect paired samples in preterm infants both after birth, and after a prolonged stay on the neonatal intensive care unit. Hair cortisol, but not hair cortisone, was associated within paired neonatal samples taken after birth and at 6 weeks. Consistent with a previous study that demonstrated hair cortisol concentrations in infants who experienced a prolonged stay within a neonatal intensive care unit where higher than those of healthy infants discharged home, hair glucocorticoids appear to reflect physiological stressors as is evidenced by the higher rate of cortisol in hair of infants who had neonatal sepsis (Yamada et al., 2007). Hair cortisol concentrations were also higher in infants who had received a 10-day course of prophylactic lose dose hydrocortisone, which is a treatment estimated to increase serum cortisol levels at the time of therapy (Watterberg et al., 2004).

The higher hair cortisol observed in infants exposed to chorioamnionitis is consistent with a previous study showing higher serum cortisol concentrations in these infants (Watterberg et al., 1997) and is suggestive that chorioamnionitis is associated with upregulation of the newborn HPA axis. This is of clinical interest given that infants exposed to chorioamnionitis are at greater risk brain dysmaturation (Anblagan et al., 2016, Sullivan et al., 2020), and neurodevelopmental abnormalities (Galinsky et al., 2013).

Strengths of this study are that hair samples were collected from the largest collection of preterm infants to date, hair cortisol and cortisone were measured using a robust LC-MS method and placental inflammation was assessed by a perinatal pathologist. Limitations include the fact that serial assessments of serum or saliva cortisol was not conducted to validate 6-week hair cortisol concentrations. Additionally, hair was not sampled from the same scalp region in all infants. However, to date variation of infant hair cortisol across different scalp areas has not been established. In this study, we sampled up to 2 cm of hair at 6 weeks. It may be that restricting measurement to the 1 cm closest to the scalp would more precisely reflect postnatal compared to prenatal exposures. To our knowledge hair growth rates in preterm infants are not currently known. Future research measuring hair growth during the neonatal period, testing variation in glucocorticoid concentrations across the length of hair, and across scalp regions, could help optimize the use of neonatal hair sampling as a marker of postnatal glucocorticoid exposure.

The average age of mothers in the term group was three years older than the Scottish average (Lowe, 2020), and there was a high proportion of twin births and male fetuses included within the preterm group. Higher maternal age has previously been associated with a lower maternal serum cortisol concentration in pregnancy (Bleker et al., 2017), and fetal sex has been associated with placental glucocorticoid metabolism (Mericq et al., 2009), fetal cortisol production (Wynne-Edwards et al., 2019) and the HPA axis of preterm infants (Grunau et al., 2010). These factors could therefore influence the generalisability of results.

## 5 Conclusions

In summary, cortisol and cortisone measured in hair sampled after birth are higher in term than preterm born infants. Neonatal hair sampled after birth likely reflects amniotic fluid glucocorticoid concentrations, and offer potential insight into how glucocorticoid metabolism varies with spontaneous labour and chorioamnionitis. Hair glucocorticoids measured at 6 weeks likely reflect both prenatal and postnatal stressors.

## Funding

The work was funded by Theirworld, UK (www.theirworld.org) and was undertaken in the MRC Centre for Reproductive Health, which is funded by 10.13039/501100000265MRC Centre, UK Grant (MRC G1002033). RMR acknowledges the support of the British Heart Foundation (RE/18/5/34216).

## Data availability

A data access and collaboration policy sets out the terms and conditions on which de-identified participant data is accessible to the research community following reasonable request.

[http://www.tebc.ed.ac.uk].

## Author contributions

D.Q.S conceived and designed the study, acquired and analysed data, and drafted the article. G.S, G.J.L and G.S.B: acquired data and revised the article critically for important intellectual content. P.G analysed data and revised the article critically for important intellectual content. C.K provided the analysis of hair samples and revised the article critically for important intellectual content. M.J.E provided the analysis of placenta samples and revised the article critically for important intellectual content. J.P.B and R.M.R conceived and designed the study and revised the article critically for important intellectual content.

## Declarations of interest

The authors have no competing interests to declare.
